# Presentation and progression of MPO-ANCA interstitial lung disease

**DOI:** 10.1016/j.jtauto.2024.100235

**Published:** 2024-02-23

**Authors:** Lorenzo Salvati, Boaz Palterer, Elena Lazzeri, Emanuele Vivarelli, Marina Amendola, Marco Allinovi, Leonardo Caroti, Alessio Mazzoni, Laura Lasagni, Giacomo Emmi, Edoardo Cavigli, Marco Del Carria, Linda Di Pietro, Mariangela Scavone, Daniele Cammelli, Federico Lavorini, Sara Tomassetti, Elisabetta Rosi, Paola Parronchi

**Affiliations:** a Department of Experimental and Clinical Medicine, University of Florence, Florence, Italy; b Department of Clinical and Experimental Biomedical Sciences, Excellence Centre for Research, Transfer and High Education for the Development of DE NOVO Therapies (DENOTHE), University of Florence, Florence, Italy; c Pneumology and Intensive Care Unit, Careggi University Hospital, Florence, Italy; d Nephrology Unit, Careggi University Hospital, Florence, Italy; e Flow Cytometry Diagnostic Center and Immunotherapy, Careggi University Hospital, Florence, Italy; f Department of Emergency Radiology, Careggi University Hospital, Florence, Italy; g Interventional Pulmonology Unit, Careggi University Hospital, Florence, Italy; h Immunology and Cell Therapy Unit, Careggi University Hospital, Florence, Italy

**Keywords:** ANCA-Associated vasculitis, Interstitial lung disease, Glomerulonephritis, MPO, UIP, MPO-ANCA, C3 deposits, Pulmonary fibrosis

## Abstract

The association between MPO-ANCA-associated vasculitis (AAV) and interstitial lung disease (ILD) has been well established. Pulmonary fibrosis may coexist with, follow, or even precede the diagnosis of AAV, and its presence adversely affects the prognosis. The optimal approach to investigating ANCA in patients with ILD remains a subject of ongoing debate. Here we aim to describe presentation and progression of MPO-ANCA ILD. We conducted a retrospective evaluation of a cohort of individuals diagnosed with MPO-ANCA ILD, with or without accompanying renal impairment, at the Immunology and Cell Therapy Unit, Careggi University Hospital, Florence, Italy, between June 2016 and June 2022. Clinical records, imaging studies, pathologic examinations, and laboratory test results were collected. Among the 14 patients identified with MPO-ANCA ILD, we observed a significant association between MPO-ANCA titers assessed at the time of ILD diagnosis and renal involvement. Renal impairment in these cases often manifested as subclinical or slowly progressive kidney damage. Interestingly, complement C3 deposits were consistently found in all renal biopsy specimens, thereby suggesting the potential for novel therapeutic targets in managing renal complications associated with MPO-ANCA ILD. The presentation of MPO-ANCA vasculitis as ILD can be the first and only clinical manifestation. MPO-ANCA levels at ILD diagnosis could warn on the progression to renal involvement in patients with MPO-ANCA ILD, hence caution is needed because renal disease can be subclinical or smoldering.

## Abbreviations

AAV: ANCA-associated vasculitis
ANCA: Anti-neutrophil cytoplasmic antibody
EGPA: Eosinophilic granulomatosis with polyangiitis
GPA: Granulomatosis with polyangiitis
HRCT: high-resolution computed tomography
ILD: Interstitial lung disease
IPF: Idiopathic pulmonary fibrosis
MPA: Microscopic polyangiitis
MPO: Myeloperoxidase
NSIP: non-specific interstitial pneumonia
UIP: usual interstitial pneumonia
PR3: Proteinase 3

## 1 Introduction

Anti-neutrophil cytoplasmic antibodies (ANCA)-associated vasculitis (AAV) is a heterogeneous group of multiorgan autoimmune vasculitides affecting small vessels and includes three distinct syndromes: microscopic polyangiitis (MPA), granulomatosis with polyangiitis (GPA), and eosinophilic granulomatosis with polyangiitis (EGPA) [1,2]. ANCA are autoantibodies that target specific antigens found in the cytoplasmic granules of neutrophils and lysosomes of monocytes. They commonly recognize leukocyte proteinase 3 (PR3-ANCA) or myeloperoxidase (MPO-ANCA) [3]. Epidemiologic, clinical, and genetic findings suggest that AAV should be re-classified as MPO-positive AAV, PR3-positive AAV, and with regards of EGPA, accordingly to the presence or absence of MPO-ANCA [4,5]. Lung involvement is a common clinical feature of AAV, often presenting as dry cough, shortness of breath, wheezing, or hemoptysis and including necrotizing granulomatous inflammation, tracheobronchial inflammation, pulmonary capillaritis leading to diffuse alveolar hemorrhage, asthma, and interstitial lung disease (ILD) [[6], [7], [8]]. The association between AAV, almost exclusively MPO-AAV, and ILD has been known since at least 1990 and it has been increasingly recognized [[9], [10], [11], [12], [13]]. Pulmonary fibrosis may coexist, follow, or even precede the diagnosis of overt AAV and has negative impact on long-term prognosis [[14], [15], [16]]. Additionally, patients with ILD and ANCA-positivity without signs of systemic vasculitis have been reported, as well as patients that turn ANCA positive over the course of ILD. Clinical features compatible with MPA diagnosis develop in up to 25% MPO-ANCA-positive patients whose ILD was initially classified as idiopathic pulmonary fibrosis (IPF) [[17], [18], [19], [20]]. However, there is still debate over whether ANCA should be investigated systematically or on a case-by-case basis in the evaluation of patients with suspected IPF [[21], [22], [23], [24], [25], [26], [27], [28], [29]]. The spectrum of ANCA-associated ILD, particularly MPO-ANCA ILD, and the extent of immune dysregulation thus remain subjects that require further elucidation. Characterization of patients with MPO-ANCA ILD is essential to improve the diagnostic process and decide the most appropriate timing and type of treatment for these patients. Herein, we report on a cohort of patients with MPO-ANCA ILD who were followed at a single medical center, and we describe the presentation and progression of the disease.

## 2 Methods

### 2.1 Study population

This is a retrospective longitudinal monocentric cohort study. We retrospectively investigated all adult patients admitted at the Immunology and Cell Therapy Unit, Careggi University Hospital, Florence, Italy from June 2016 to June 2022 who were diagnosed as having ILD and positive ANCA testing. Last follow-up date was the latest available up to January 2024. In the context of the local multidisciplinary group for ILD, the Immunology and Cell Therapy Unit is dedicated to classification and management of adult patients with ILD and an underlying or suspected immunologic cause.

### 2.2 Data collection

Clinical data, laboratory tests, imaging studies, histopathological examinations, treatments, and outcome were retrospectively extracted from electronic medical records. Patient demographic characteristics, age at onset of pulmonary symptoms and, if present, of renal manifestations, and the time interval between lung and kidney involvement were evaluated. ANCA were detected by indirect immunofluorescence technique (IIF) using a commercial kit (EUROIMMUN, Lübeck, Germany); serum anti-MPO and anti-PR3 IgG antibodies were measured using a commercial fluorescence enzyme immunoassay (EliA, Phadia, Termo Fisher Scientific, Waltham, Massachusetts, United States). ANCA detection (IIF/FEIA) was performed at ILD diagnosis. The presence of ILD was assessed by chest high-resolution computed tomography (HRCT) scans. Lung and kidney biopsy specimens, when available, were retrieved and reviewed. Renal involvement was defined as the presence of glomerulonephritis on kidney biopsy specimens and/or laboratory tests alterations suggestive of kidney damage, irrespective of the time of ILD diagnosis. Direct immunofluorescence was performed on fresh frozen renal biopsy specimens. The intensity of immunostaining was scored as negative (−), weak (±), mild (1+), moderate (2+), and strong (3+) [30].

### 2.3 Statistical analysis

Continuous variables are expressed as median and interquartile range (IQR) or mean and standard deviation (SD), while categorical variables expressed as number (%). Mann-Whitney *U* test was used to assess variations in continuous variables. p value less than 0.05 was considered statistically significant. Data were analyzed using OriginPro, Version 95E (OriginLab Corporation, Northampton, MA, USA).

## 3 Results

### 3.1 Patient characteristics

**Fig. 1:**
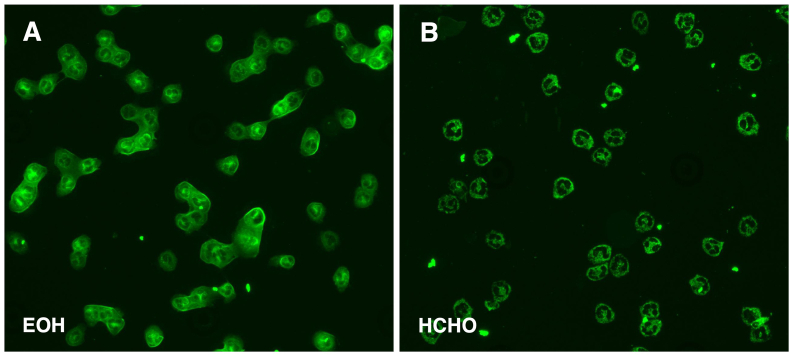
**ANCA indirect immunofluorescence (IIF) pattern.** The serum analysis of a 64-year-old woman with interstitial lung disease and new onset kidney disease revealed a peri-nuclear (P)-ANCA pattern on ethanol-fixed granulocytes (A) that shifted into a cytoplasmic pattern on formalin-fixed cells (B). This particular pattern is indicative of positive anti-myeloperoxidase (MPO) antibodies. The patient was found to have elevated anti-MPO antibodies titers. EOH: neutrophils fixation in ethanol; HCOH: neutrophils fixation in formaldehyde.

Over a six-year period from June 2016 to June 2022, a total of 14 patients with ILD and positive ANCA were diagnosed at the Immunology and Cell Therapy Unit, Careggi University Hospital in Florence, Italy (Table 1 and Supplementary Table 1). Out of these patients, 8 were female (aged 70.5 years, IQR 59.5–73.5), while 6 were male (aged 63.5 years, IQR 56.3–70.8). The difference in age between the two genders was not found to be statistically significant (*p* = 0.366). Among the patients, 79% were ex-smokers, with an average smoking history of 19 pack-years (Table 1). Three patients (21%) had a positive family history of fibrotic lung disease. Notably, two of these patients also presented with premature graying of hair. All patients showed IIF-ANCA perinuclear pattern (P-ANCA) at diagnosis (Fig. 1). Anti-MPO antibodies were present in all the cases with a median titer at ILD diagnosis of 34.5 UI/mL (IQR 11–111).

**Table 1:** Demographic and clinical characteristics of 14 patients with MPO-ANCA ILD.

Variable	n (%)
Gender, n (%)	–
Male	6 (43%)
Female	8 (57%)
Ethnicity, n (%)	–
Caucasian	14 (100%)
Smoking history, n (%)	–
No smokers	3 (21%)
Previous smokers (pack-years, mean ± SD)	11 (79%) (19 ± 17)
Active smokers	0
Exposure history, n (%)	2 (14%)^a^
Age at first immunology visit, yrs (mean ± SD)	66 ± 8
Age at pulmonary symptoms/signs onset, yrs (mean ± SD)	62 ± 9
Pulmonary symptoms at onset, n (%)	–
- Dyspnea	10 (71%)
- Dry cough	7 (50%)
- Dyspnea + dry cough	5 (36%)
- Incidental finding	2 (14%)
HRCT pattern, n (%)	–
- UIP	8 (57%)
- Possible UIP	1 (7%)
- NSIP	3 (21%)
- Bronchiolitis	2 (14%)
Age at renal symptoms/signs onset, yrs (mean ± SD)	65 ± 6
Renal involvement, n (%)	8 (57%)

a Asbestos, parrots.

### 3.2 Pulmonary involvement

Exertional dyspnea (10/14, 71%), dry cough (7/14, 50%) which was frequently associated with dyspnea (5/7, 71%) were the most common presentations of ILD. Incidental lung abnormalities on imaging were only found in 2 patients. One case was identified during a screening chest imaging procedure prompted by a family history of IPF, while the other was detected after a CT scan of the abdomen performed for a complaint of lower back pain. The age at onset of pulmonary manifestations was 66 years (IQR 54–69), 60 years (IQR 51–68) for males and 67 years (IQR 55–70) for females. HRCT findings were consistent with usual interstitial pneumonia (UIP) pattern in the majority of cases, specifically 57% (8/14). Non-specific interstitial pneumonia (NSIP) was observed in 3 cases, a possible UIP pattern in 1 case, and bronchiolitis in 2 cases (Table 1). Lung biopsies were performed in three patients to assess the specific pattern of the disease and revealed in one case (Patient 5) a generally preserved pulmonary architecture, alternating normal lung tissue areas to fibrotic thickening of the interstitium, often accompanied by an inflammatory infiltrate mainly composed of lymphocytes and plasma cells compatible with a final diagnosis of NSIP. In Patient 13 the histologic examination of the bronchial mucosa and lung parenchyma showed a lymphoid infiltrate, diffusely and perivascularly distributed, comprising both B and T cells, along with numerous polyclonal plasma cells. No evidence of granulomas or features indicative of leukocytoclastic vasculitis were observed. In contrast, in Patient 14 non-necrotizing granulomatous inflammation of the bronchial mucosa and lung parenchyma associated with an intense mixed-type inflammatory infiltrate mainly involving the lung interstitium was revealed. The final diagnosis has been made on the basis of the histopathology pattern.

### 3.3 Renal involvement

**Fig. 2:**
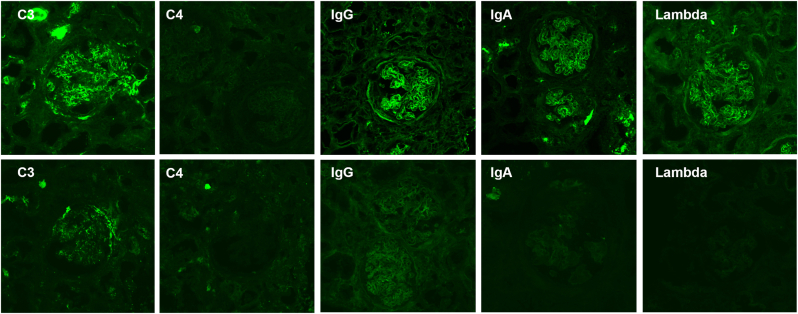
**Immunofluorescence staining (C3, C4, IgG, IgA, lambda chains) of kidney biopsy samples from two patients with MPO-ANCA ILD.** Immune deposits of C3 are observed in both patients, with higher intensity in Patient 1 (upper row) compared to Patient 2 (lower row). Patient 1 (upper row) underwent kidney biopsy concurrently with the onset of renal function deterioration. The images show immune deposits of C3 with a high intensity (+++), along with deposits of IgG (++), IgA (++) and Lambda chains (++), but no C4 deposits. Conversely, Patient 2 (lower row) had a longer history of kidney disease before undergoing the biopsy. In this case, predominant interstitial fibrosis was observed on light microscopy, and the direct immunofluorescence staining showed only C3 deposition.

More than one half of the MPO-ANCA-positive patients (8/14 cases) developed renal involvement. The median age at onset of renal manifestations was 68 years (IQR 61–70), 68 (IQR 58–70) for males and 66 (IQR 59–70) for females. Renal manifestations more frequently occurred after pulmonary symptom onset (75%, 6/8 cases) than concomitantly (12.5%, 1/8 cases) or before (12.5%, 1/8 cases). The interval between the pulmonary symptom onset and the appearance of renal manifestations, ranged from 13 to 125 months, with a median of 38 months (IQR 22–60). Only in one case out of 8, renal impairment anticipated ILD. Urinary manifestations were more commonly non-nephrotic proteinuria and hematuria. Six out of the 8 patients with renal involvement underwent kidney biopsy. The histological findings were consistent with glomerulonephritis, as reported in Supplementary Table 1. Direct immunofluorescence of the kidney biopsies revealed the deposition of C3 complement in both parietal and capillary regions. The intensity of C3 deposition varied, ranging from weak to strong, but it was present in all the specimens (Supplementary Table 1). In Fig. 2 immunofluorescence staining of kidney biopsy samples from two patients with MPO-ANCA ILD is presented.

### 3.4 High anti-MPO titers associate with renal involvement in MPO-ANCA ILD

**Fig. 3:**
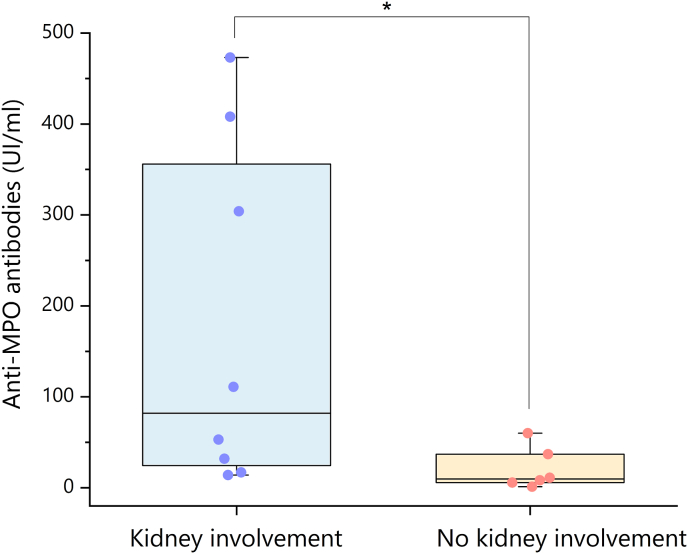
**Anti-MPO antibodies titers stratified by renal involvement in patients with MPO-ANCA ILD.** Anti-MPO antibodies titers (measured in UI/mL by a commercial ELISA) were evaluated at the time of ILD diagnosis in patients with MPO-ANCA ILD with (n = 8, blue circles) and without (n = 6, orange circles) renal involvement. Box plots represent 25th to 75th percentiles. Black line inside the box represents the median. Whiskers represent maximum and minimum values. *p < 0.05; calculated with Mann-Whitney *U* test.

**Fig. 4:**
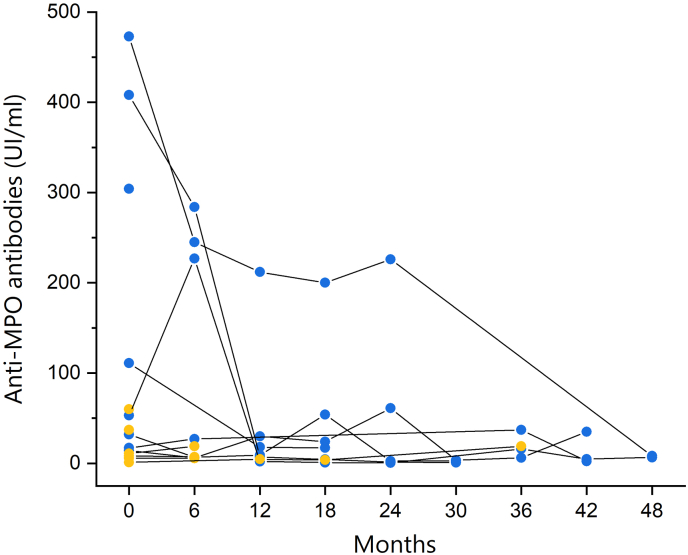
**Longitudinal evaluation of anti-MPO antibodies titers.** Where available, anti-MPO antibodies titers were retrieved up to 48 months after detection at ILD diagnosis in patients with MPO-ANCA ILD with (n = 8, blue circles) and without (n = 6, orange circles) renal involvement.

Patients with renal involvement had significantly higher MPO-ANCA titers at lung disease onset (82 UI/mL, IQR 20.8–382) when compared to patients without renal impairment (9.6 UI/mL, IQR 4.6–42.8) (*p* = 0.016) (Fig. 3). Considering only MPO-ANCA ILD patients that eventually developed kidney disease, median MPO-ANCA titers at lung disease onset were 53 UI/mL (IQR 24.5–259.5), without statistically relevant difference (*p* = 0.0828) when compared to subjects without subsequent renal disease. Where available, additional evaluations of MPO-ANCA titers were retrieved showing reduction over time according to initiation of immunosuppressive/immunomodulatory treatment (Fig. 4).

### 3.5 Treatment and outcomes

Patients with renal impairment, in addition to courses of prednisone, received mycophenolate mofetil (1000 mg twice daily). Rituximab (two initial doses of 1000 mg each, given 2 weeks apart, followed by a third dose at month 3, and then every 6 months throughout a 2-year treatment cycle) was administered to 4 patients. Anti-fibrotic treatment was used in 5 patients (pirfenidone n = 4, nintedanib n = 1). During the follow-up period (median 53 months, IQR 40–64), 4 out of 14 patients died of respiratory failure (one because of SARS-CoV-2 infection). Deceased patients had renal involvement in 50% of cases. A total of 3 patients were lost to follow-up. A total of 6 patients with MPO-ANCA ILD did not manifest signs of renal involvement at a median follow-up time of 57 months (IQR 15–61). Notably, 2 out of 6 patients were monitored for a duration shorter than the median interval from ILD onset to kidney disease manifestation as observed in the subgroup of patients with kidney involvement. During the study period, no patient developed GPA or EGPA.

## 4 Discussion

The spectrum of MPO-ANCA positive interstitial pneumonia is multifaceted, from isolated lung involvement to pulmonary-renal syndrome. Notably, up to a quarter of ANCA-positive IPF patients will eventually develop MPA [[17], [18], [19], [20],^31^], highlighting the complex nature of MPO-ANCA ILD. MPA typically presents with constitutional symptoms and organ-specific manifestations, often with elevated inflammatory markers. Renal involvement is common and rapidly progressive glomerulonephritis is the major clinical feature of MPA [32,33]. Patients with MPO-ANCA ILD have primarily lung involvement. Although UIP pattern is the most common imaging finding, as observed in IPF patients, other presentations are reported, varying from NSIP to unclassifiable IIP [19,31,34,35]. Renal involvement may also occur, but it is slowly-progressive and far to be as pronounced as in MPA, so that it can be overlooked. In our retrospective study, 57% of MPO-ANCA ILD patients had renal involvement and 50% of those without initial renal involvement developed glomerulonephritis diagnosed at a median time of 3.2 years after the onset of pulmonary symptoms, with a maximum interval of 10 years. This finding holds crucial implications for clinical practice. Firstly, it underscores the importance of vigilant monitoring and early detection of renal damage, especially in patients initially classified as having IPF. In fact, timely identification is of paramounting relevance for appropriate management and therapeutic interventions before irreversible renal damage occurs. Secondly, in previous guidelines for diagnosing IPF, ANCA testing was included only when vasculitis was suspected [21], but MPO-ANCA ILD tend to present without common signs of vasculitis. Interestingly, an analysis of the World Health Organization pharmacovigilance database (VigiBase) found nintedanib to be associated with AAV [36]. Actually, it is possible that overreporting of this association occurred due to misdiagnosis of IPF (treated with nintedanib) with unrecognized AAV-ILD at its onset [36]. On the other hand, the 2022 ACR/EULAR classification criteria for MPA give relevant weight to ILD with a score of 9, resulting the second strongest independent variables for MPA classification [37]. However, it is still debated whether MPO-ANCA ILD, without additional organ damage associated with AAV besides interstitial pneumonia, should fall under AAV. More than half of our patients developed renal disease, mainly after pulmonary symptoms onset. Studies in Japan and North America also show a progression towards MPA in some MPO-ANCA ILD (from 22% to 35% of cases) [17,19,20,38]. Nonetheless, the renal involvement is far less pronounced compared to MPA. Viewed from the opposite perspective, in a cohort of 41 patients with slowly-progressive AAV-renal vasculitis, Trivioli et al. showed that 24% had interstitial lung lesions [39]. Notably, in 4 patients who had not previously undergone chest HRCT, pulmonary fibrosis was found during follow-up [39]. A comparison of the present study with previously published reports of MPO-ANCA ILD patients is summarized in Table 2 [17,19,20,34,35,38,[40], [41], [42], [43], [44], [45], [46]].

**Table 2:** Baseline characteristics of current patients compared with main series describing MPO-ANCA ILD.

Country	Report, Year [PMID]	Patients, n	Gender (M/F)	Age (median, years)	Smoking history	UIP pattern on HRCT	Renal involvement
Italy	Current case series, 2024	14	6/8	68.5	11 (79%)	8 (57%)	8 (57%)
Italy	Sebastiani, 2021 [34207641]	58	27/31	69	23 (39%)	30 (51.7%)	0
Japan	Yamaguchi, 2021 [34056682]	63	27/36	76	30 (48%)	na	34 (54%)
US	Liu, 2019 [31181198]	18	6/12	67.5	na	na	3 (17%)
US	Baqir, 2019 [ 32476954]	18	10/8	58	10 (56%)	4 (22%)	na
Japan	Hozumi, 2018 [29928060]	15	12/3	68	13 (87%)	9 (60%)	6 (40%)
Japan	Hosoda, 2016 [26994375]	12	8/4	65.2	6 (50%)	12 (100%)	3 (25%)
Japan	Kagiyama, 2015 [25593704]	20	11/9	71	11 (55%)	na	6 (30%)
Argentina	Fernandez Casares, 2015 [24863847]	9	5/4	64	7 (78%)	6 (66%)	6 (66%)
China	Huang, 2014 [24468083]	19	8/11	66	na	19 (100%)^b^	12 (63%)
Japan	Ando, 2013 [23434037]	9	9/0	69	9 (100%)	9 (100%)^b^	2 (22%)
UK	Arulkumaran, 2011 [21873269]	14	10/4	67.5	10 (71%)	8 (57%)	14 (100%)
France	Hervier, 2009 [18957485]	12	9/3	70.5	8 (67%)	6 (50%)^a^	8 (67%)
France	Foulon, 2008 [18640019]	6	6/0	65.5	4 (67%)	na	4 (67%)

Na denotes not available.

a Data not available for all patients.

b Includes possible UIP pattern.

A second interesting point is the significant association between higher anti-MPO antibody titers at the onset of pulmonary symptoms and the development of kidney disease in patients with MPO-ANCA ILD. These findings are consistent with previous studies demonstrating significantly higher MPO-ANCA titers in patients with ILD in the context of MPA compared to ANCA-positive IPF patients [47] and in patients with MPO-AAV who have poor initial renal function and worse renal outcomes [48]. MPO-AAV frequently results in severe glomerulonephritis. In murine models, it has been demonstrated that lower doses of MPO-ANCA induce glomerular leukocyte adhesion only after an infection-related stimulus, while higher antibody doses can induce it independently through alternative adhesion mechanisms [49]. ANCAs bind to and prime neutrophils [50]. During their migration through the high shear stress glomerular capillary network, neutrophils undergo degranulation, releasing NETs [51,52]. This process induces microvascular endothelial injury driven by the activation of the complement pathway [51]. However, also MPO-ANCA affinity and specificity is relevant. Yoshida et al. demonstrated that MPA patients with high affinity MPO-ANCA mainly presented with rapidly progressive glomerulonephritis and histological findings consistent with focal/crescentic glomerulonephritis, whereas MPA patients with low affinity MPO-ANCA mainly displayed clinical signs of chronic renal failure and mixed/sclerotic glomerulonephritis [53]. Interestingly, patients with high affinity antibodies had a significantly higher incidence of NETs compared to patients with low affinity antibodies, suggesting that the affinity of ANCA is associated with the in vivo formation of NETs [53]. The mapping of MPO-ANCA epitopes unveiled a substantial number of MPO-ANCA/epitope combinations, which have been associated to disease activity, or naturally occurring in healthy subjects [[53], [54], [55], [56], [57]]. Diagnostic utility of ANCA is well-established, but clinical utility of serial ANCA testing remains controversial to monitor disease activity [58]. Larger-scale studies are needed to fully establish MPO-ANCA titers as a potential prognostic biomarker in patients with MPO-ANCA ILD, but serological monitoring might be useful in predicting renal involvement in MPO-ANCA ILD patients and of aid in risk stratification. In patients with MPO-AAV and glomerulonephritis, it has been recently described that persistence or subsequent reappearance of MPO-ANCA is associated with a higher risk of relapse, while MPO-ANCA negativity stratifies a low risk for relapse even without remission maintenance therapy [59].

Finally, we found glomerular and parietal C3 deposits in kidney biopsies of MPO-ANCA ILD patients. This challenges the historic view of AAV as pauci-immune because of minimal antibody and complement deposition in the glomerulus on kidney biopsy [51]. The presence of C3 deposits have been associated with a worse outcome in ANCA glomerulonephritis [[60], [61], [62]] and complement activation, particularly the alternative pathway, has emerged as being crucial in the development of AAV [63]. Animal studies support these observations, showing that anti-MPO IgGs induce kidney damage with C3 deposits [63], while complement-depleted mice are protected from kidney damage [64]. C3 represents the most common complement component found in MPO-ANCA-associated glomerulonephritis [60,[65], [66], [67]]. A proteomic analysis revealed elevated levels of complement proteins, primarily C3, in glomeruli affected by crescents and necrosis when compared to normal glomeruli in patients with MPO-ANCA-associated glomerulonephritis [68]. Complement-mediated inflammation seems pivotal in ANCA glomerulonephritis: the C5 cleavage product C5a acts as an anaphylatoxin engaging C5a receptors on neutrophils, promoting their activation and chemoattraction [[69], [70]]. C5aR blockade via avacopan – an oral small-molecule C5a receptor antagonist – has proven to be effective and recently introduced in the treatment of AAV [[71], [72], [73]]. Unfortunately, neither circulating immune complexes (CICs) nor soluble complement levels were evaluated in our cohort. Nonetheless, no relation between CICs and complement components glomerular deposition was found by Hilhorst et al. [66].

**Fig. 5:**
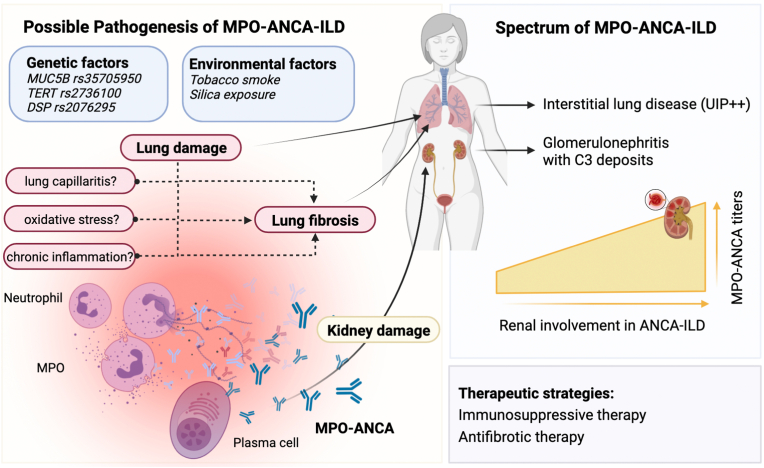
**Potential pathogenesis and clinical spectrum of patients with MPO-ANCA ILD.** Genetic and environmental factors may contribute to susceptibility. Lung damage may result from endothelial inflammation, oxidative stress, and chronic inflammation leading to fibrosis. Autoimmunity arises due to loss of T and B cell tolerance, with B cells producing ANCA targeting neutrophils. This activates neutrophils, causing vessel damage. It is not known whether MPO-ANCA are needed for pulmonary damage. It could be hypothesized that the immune response originates in the lung, then autoantibodies are directly pathogenic in the kidney. Consequently, there is a linear correlation between kidney damage and antibody titer. Clinical spectrum ranges from isolated fibrotic lung disease to AAV-associated ILD with varying degrees of renal involvement. Therapeutic strategies include immunosuppression and anti-fibrotic treatments. (Illustration created with BioRender.com).

A common pathogenic sequence in MPO-ANCA ILD could be envisaged (Fig. 5), involving genetic and environmental factors leading to lung damage through neutrophilic inflammation [35], subsequent autoimmunity, and fibrosis [[74], [75], [76]]. While C3 deposits are present in the kidney, it is unclear if complement-driven inflammation affects pulmonary pathogenesis. Avacopan might hold promise if MPO-ANCA ILD is complement-mediated. Empirical management approach to patients with MPO-ANCA ILD has been proposed [8,77,78] and there are no guidelines, apart from patients with overt MPA [79].

This study is limited by the small sample size, which is due to the monocentric and observational nature of the study as well as the rarity of the condition. Moreover, within the non-kidney involvement subgroup, some patients were monitored for a duration shorter than the median interval from ILD onset to kidney disease manifestation as observed in the kidney subgroup.

In conclusion, our findings, though from a case-series, align with prior observations, highlighting the diverse spectrum of MPO-ANCA ILD. The role of MPO-ANCA in the pathogenesis of ILD is still a subject of ongoing discussion. However, their presence strongly indicates a potential pathogenetic pulmonary-renal continuum, with higher levels at ILD diagnosis associated with subsequent kidney disease. Further research is needed to better understand the underlying pathogenic mechanisms and implications of MPO-ANCA in the context of MPO-ANCA ILD and its associated renal involvement.

## Funding

None.

## Human ethics approval declaration

This is a retrospective study designed to analyze historical patient data from routine clinical practice. As such, it does not involve any additional or unique investigational procedure beyond what is considered standard practice in our healthcare facility. The primary aim of this study is to gain insights and draw conclusions from the existing patient records. Given the retrospective and non-interventional nature of this study, it was not necessary to assign a specific study identifier. Established ethical and regulatory guidelines were followed, and no prospective research involving new interventions, treatments, or experimental protocols was done. All participants provided informed consent to the study.

## CRediT authorship contribution statement

**Lorenzo Salvati:** Writing – review & editing, Writing – original draft, Visualization, Validation, Methodology, Investigation, Formal analysis, Data curation, Conceptualization. **Boaz Palterer:** Writing – review & editing, Writing – original draft, Visualization, Validation, Supervision, Methodology, Formal analysis, Data curation, Conceptualization. **Elena Lazzeri:** Writing – review & editing, Visualization, Validation, Formal analysis, Data curation. **Emanuele Vivarelli:** Writing – review & editing, Visualization, Investigation. **Marina Amendola:** Writing – review & editing, Validation, Investigation. **Marco Allinovi:** Writing – review & editing, Validation, Investigation. **Leonardo Caroti:** Writing – review & editing, Validation, Investigation. **Alessio Mazzoni:** Writing – review & editing, Validation, Investigation. **Laura Lasagni:** Writing – review & editing, Validation, Investigation. **Giacomo Emmi:** Writing – review & editing, Validation, Investigation. **Edoardo Cavigli:** Writing – review & editing, Visualization, Investigation. **Marco Del Carria:** Writing – review & editing, Visualization, Investigation. **Linda Di Pietro:** Writing – review & editing, Visualization, Investigation. **Mariangela Scavone:** Writing – review & editing, Visualization, Investigation. **Daniele Cammelli:** Writing – review & editing, Visualization, Supervision. **Federico Lavorini:** Writing – review & editing, Validation, Supervision, Investigation. **Sara Tomassetti:** Writing – review & editing, Validation, Supervision, Investigation. **Elisabetta Rosi:** Writing – review & editing, Visualization, Validation, Investigation. **Paola Parronchi:** Writing – review & editing, Writing – original draft, Visualization, Validation, Supervision, Methodology, Conceptualization.

## Declaration of competing interest

The authors declare that they have no known competing financial interests or personal relationships that could have appeared to influence the work reported in this paper.

## Data Availability

Data will be made available on request.

## References

[1] Jennette J.C., Falk R.J., Bacon P.A., Basu N., Cid M.C., Ferrario F., Flores-Suarez L.F., Gross W.L., Guillevin L., Hagen E.C., Hoff-man G.S., Jayne D.R., Kallenberg C.G., Lamprecht P., Langford C.A., Luqmani R.A., Mahr A.D., Matteson E.L., Merkel P.A., Ozen S., Pusey C.D., Rasmussen N., Rees A.J., Scott D.G., Specks U., Stone J.H., Takahashi K., Watts R.A. (2012). Revised international Chapel Hill consensus Conference Nomenclature of vasculitides. Arthritis Rheum..

[2] Kitching A.R., Anders H.J., Basu N., Brouwer E., Gordon J., Jayne D.R., Kullman J., Lyons P.A., Merkel P.A., Savage C.O.S., Specks U., Kain R. (2020 Aug 27). ANCA-associated vasculitis. Nat. Rev. Dis. Prim..

[3] Cornec D., Cornec-Le Gall E., Fervenza F.C., Specks U. (2016 Oct). ANCA-associated vasculitis - clinical utility of using ANCA speci-ficity to classify patients. Nat. Rev. Rheumatol..

[4] Millet A., Pederzoli-Ribeil M., Guillevin L., Witko-Sarsat V., Mouthon L. (2013 Aug). Antineutrophil cytoplasmic antibody-associated vasculitides: is it time to split up the group?. Ann. Rheum. Dis..

[5] Lyons P.A., Peters J.E., Alberici F., Liley J., Coulson R.M.R., Astle W., Baldini C., Bonatti F., Cid M.C., Elding H., Emmi G., Epplen J., Guillevin L., Jayne D.R.W., Jiang T., Gunnarsson I., Lamprecht P., Leslie S., Little M.A., Martorana D., Moosig F., Neumann T., Ohlsson S., Quickert S., Ramirez G.A., Rewerska B., Schett G., Sinico R.A., Szczeklik W., Tesar V., Vukcevic D., European Vas-culitis Genetics Consortium, Terrier B., Watts R.A., Vaglio A., Ju Holle, Wallace C., Smith K.G.C. (2019 Nov 12). Genome-wide association study of eosinophilic granulomatosis with polyangiitis reveals genomic loci stratified by ANCA status. Nat. Commun..

[6] Makhzoum J.P., Grayson P.C., Ponte C., Robson J., Suppiah R., Watts R.A., Luqmani R., Merkel P.A., Pagnoux C., collab-orators D.C.V.A.S. (2021 Mar 31). Pulmonary involvement in primary systemic vasculitides. Rheumatology.

[7] Sacoto G., Boukhlal S., Specks U., Flores-Suárez L.F., Cornec D. (2020 Oct). Lung involvement in ANCA-associated vasculitis. Presse Med..

[8] Moura M.C., Specks U. (2020). Rare Diseases of the Immune System.

[9] Nada A.K., Torres V.E., Ryu J.H., Lie J.T., Holley K.E. (1990 Jun). Pulmonary fibrosis as an unusual clinical manifestation of a pulmo-nary-renal vasculitis in elderly patients. Mayo Clin. Proc..

[10] Katsumata Y., Kawaguchi Y., Yamanaka H. (2015 Sep 23). Interstitial lung disease with ANCA-associated vasculitis. Clin Med In-sights Circ Respir Pulm Med.

[11] Streho M., Sable-Fourtassou R., Brion M.C., Bourotte I., Valeyre D., Brauner M., Dhote R., Abad S. (2006 Sep). Syndrome de Churg-Strauss et fibrose pulmonaire: une association exceptionnelle [Churg-Strauss syndrome and pulmonary fibrosis: an unusual association]. Presse Med..

[12] Comarmond C., Crestani B., Tazi A., Hervier B., Adam-Marchand S., Nunes H., Cohen-Aubart F., Wislez M., Cadranel J., Housset B., Lloret-Linares C., Sève P., Pagnoux C., Abad S., Camuset J., Bienvenu B., Duruisseaux M., Hachulla E., Arlet J.B., Hamidou M., Mahr A., Resche-Rigon M., Brun A.L., Grenier P., Cacoub P., Saadoun D. (2014). Pulmonary fibrosis in antineutrophil cytoplasmic antibodies (ANCA)-associated vasculitis: a series of 49 patients and review of the literature. Medicine (Baltim.).

[13] Flores-Suárez L.F., Sacoto G. (2019). Interstitial lung disease and ANCA-associated vasculitis. Curr Treat Options in Rheum.

[14] Maillet T., Goletto T., Beltramo G., Dupuy H., Jouneau S., Borie R., Crestani B., Cottin V., Blockmans D., Lazaro E., Naccache J.M., Pugnet G., Nunes H., de Menthon M., Devilliers H., Bonniaud P., Puéchal X., Mouthon L., Bonnotte B., Guillevin L., Terrier B., Samson M., French Vasculitis Study Group (FVSG) (2020). Usual interstitial pneumonia in ANCA-associated vasculitis: a poor prognostic factor. J. Autoimmun..

[15] Zhou P., Ma J., Wang G. (2021). Impact of interstitial lung disease on mortality in ANCA-associated vasculitis: a systematic lit-erature review and meta-analysis. Chron. Respir. Dis..

[16] Matsuda S., Kotani T., Suzuka T., Kiboshi T., Fukui K., Wakama M., Ishida T., Fujiki Y., Shiba H., Nagai K., Hata K., Shoda T., Ito Y., Makino S., Takeuchi T. (2021). Evaluation of poor prognostic factors of respiratory related death in microscopic polyan-giitis complicated by interstitial lung disease. Sci. Rep..

[17] Kagiyama N., Takayanagi N., Kanauchi T., Ishiguro T., Yanagisawa T., Sugita Y. (2015). Antineutrophil cytoplasmic anti-body-positive conversion and microscopic polyangiitis development in patients with idiopathic pulmonary fibrosis. BMJ Open Respir Res.

[18] Alba M.A., Flores-Suárez L.F., Henderson A.G., Xiao H., Hu P., Nachman P.H., Falk R.J., Charles Jennette J. (2017 Jul). Interstital lung dis-ease in ANCA vasculitis. Autoimmun. Rev..

[19] Hozumi H., Oyama Y., Yasui H., Suzuki Y., Kono M., Karayama M., Furuhashi K., Enomoto N., Fujisawa T., Inui N., Naka-mura Y., Suda T. (2018). Clinical significance of myeloperoxidase-anti-neutrophil cytoplasmic antibody in idiopathic interstitial pneumonias. PLoS One.

[20] Liu G.Y., Ventura I.B., Achtar-Zadeh N., Elicker B.M., Jones K.D., Wolters P.J., Collard H.R., Adegunsoye A., Strek M.E., Ley B. (2019 Oct). Prevalence and clinical significance of antineutrophil cytoplasmic antibodies in North American patients with idio-pathic pulmonary fibrosis. Chest.

[21] Raghu G., Remy-Jardin M., Myers J.L., Richeldi L., Ryerson C.J., Lederer D.J., Behr J., Cottin V., Danoff S.K., Morell F., Flaherty K.R., Wells A., Martinez F.J., Azuma A., Bice T.J., Bouros D., Brown K.K., Collard H.R., Duggal A., Galvin L., Inoue Y., Jenkins R.G., Johkoh T., Kazerooni E.A., Kitaichi M., Knight S.L., Mansour G., Nicholson A.G., Pipavath S.N.J., Buendía-Roldán I., Sel-man M., Travis W.D., Walsh S., Wilson K.C. (2018). American Thoracic Society, European respiratory society, Japanese respira-tory society, and Latin American thoracic society. Diagnosis of idiopathic pulmonary fibrosis. An official ATS/ERS/JRS/ALAT clinical practice guideline. Am. J. Respir. Crit. Care Med..

[22] Raghu G., Remy-Jardin M., Richeldi L., Thomson C.C., Inoue Y., Johkoh T., Kreuter M., Lynch D.A., Maher T.M., Martinez F.J., Molina-Molina M., Myers J.L., Nicholson A.G., Ryerson C.J., Strek M.E., Troy L.K., Wijsenbeek M., Mammen M.J., Hossain T., Bissell B.D., Herman D.D., Hon S.M., Kheir F., Khor Y.H., Macrea M., Antoniou K.M., Bouros D., Buendia-Roldan I., Caro F., Crestani B., Ho L., Morisset J., Olson A.L., Podolanczuk A., Poletti V., Selman M., Ewing T., Jones S., Knight S.L., Ghazipura M., Wilson K.C. (2022 May 1). Idiopathic pulmonary fibrosis (an update) and progressive pulmonary fibrosis in adults: an official ATS/ERS/JRS/ALAT clinical practice guideline. Am. J. Respir. Crit. Care Med..

[23] Wijsenbeek M., Cottin V. (2020 Sep 3). Spectrum of fibrotic lung diseases. N. Engl. J. Med..

[24] Moiseev S., Cohen Tervaert J.W., Arimura Y., Bogdanos D.P., Csernok E., Damoiseaux J., Ferrante M., Flores-Suárez L.F., Fritzler M.J., Invernizzi P., Jayne D., Jennette J.C., Little M.A., McAdoo S.P., Novikov P., Pusey C.D., Radice A., Salama A.D., Sa-vige J.A., Segelmark M., Shoenfeld Y., Sinico R.A., Sousa M.J., Specks U., Terrier B., Tzioufas A.G., Vermeire S., Zhao M.H., Bos-suyt X. (2020). International consensus on ANCA testing beyond systemic vasculitis. Autoimmun. Rev..

[25] Salvati L., Palterer B., Parronchi P. (2020 Dec 17). Spectrum of fibrotic lung diseases. N. Engl. J. Med..

[26] Fidler L.M., Kandel S., Fisher J.H., Mittoo S., Shapera S. (2021). Utility of anti-neutrophil cytoplasmic antibody screening in idio-pathic interstitial lung disease. Sarcoidosis Vasc. Diffuse Lung Dis..

[27] Kadura S., Raghu G. (2021 Nov 8). Antineutrophil cytoplasmic antibody-associated interstitial lung disease: a review. Eur. Respir. Rev..

[28] Pineton de Chambrun M., Nunes H., Brochériou I., Hertig A. (2015 Oct 26). Idiopathic lung fibrosis and anti myeloperoxidase glomeru-lonephritis: the tree that hides the forest. BMC Pulm. Med..

[29] Traila D., Marc M.S., Pescaru C., Manolescu D., Fira-Mladinescu O. (2022 Mar 4). ANCA-associated vasculitis in idiopathic pulmonary fibrosis: a case report and brief review of the literature. Medicine (Baltim.).

[30] Sethi S., Haas M., Markowitz G.S., D'Agati V.D., Rennke H.G., Jennette J.C., Bajema I.M., Alpers C.E., Chang A., Cornell L.D., Co-sio F.G., Fogo A.B., Glassock R.J., Hariharan S., Kambham N., Lager D.J., Leung N., Mengel M., Nath K.A., Roberts I.S., Rovin B.H., Seshan S.V., Smith R.J., Walker P.D., Winearls C.G., Appel G.B., Alexander M.P., Cattran D.C., Casado C.A., Cook H.T., De Vriese A.S., Radhakrishnan J., Racusen L.C., Ronco P., Fervenza F.C. (2016 May). Mayo clinic/renal pathology society consensus report on pathologic classification, diagnosis, and reporting of GN. J. Am. Soc. Nephrol..

[31] Bando M., Homma S., Harigai M. (2022). MPO-ANCA positive interstitial pneumonia: current knowledge and future perspec-tives. Sarcoidosis Vasc. Diffuse Lung Dis..

[32] Chung S.A., Seo P. (2010 Aug). Microscopic polyangiitis. Rheum. Dis. Clin. N. Am..

[33] Agard C., Mouthon L., Mahr A., Guillevin L. (2003 Oct 15). Microscopic polyangiitis and polyarteritis nodosa: how and when do they start?. Arthritis Rheum..

[34] Baqir M., Yi E.E., Colby T.V., Cox C.W., Ryu J.H., Specks U. (2019). Radiologic and pathologic characteristics of myeloperoxi-dase-antineutrophil cytoplasmic antibody-associated interstitial lung disease: a retrospective analysis. Sarcoidosis Vasc. Diffuse Lung Dis..

[35] Hosoda C., Baba T., Hagiwara E., Ito H., Matsuo N., Kitamura H., Iwasawa T., Okudela K., Takemura T., Ogura T. (2016 Jul). Clinical features of usual interstitial pneumonia with anti-neutrophil cytoplasmic antibody in comparison with idiopathic pulmonary fibrosis. Respirology.

[36] Deshayes S., Dolladille C., Dumont A., Martin Silva N., Chretien B., De Boysson H., Alexandre J., Aouba A. (2021 Jun 23). A worldwide pharmacoepidemiological update of drug-associated ANCA-associated vasculitis at the time of targeted therapies. Arthritis Rheumatol..

[37] Suppiah R., Robson J.C., Grayson P.C., Ponte C., Craven A., Khalid S., Judge A., Hutchings A., Merkel P.A., Luqmani R.A., Watts R.A., DCVAS INVESTIGATORS (2022). American college of rheumatology/European alliance of associations for rheu-matology classification criteria for microscopic polyangiitis. Ann. Rheum. Dis..

[38] Ando M., Miyazaki E., Ishii T., Mukai Y., Yamasue M., Fujisaki H., Ito T., Nureki S., Kumamoto T. (2013 Apr). Incidence of myeloperox-idase anti-neutrophil cytoplasmic antibody positivity and microscopic polyangitis in the course of idiopathic pulmonary fibrosis. Respir. Med..

[39] Trivioli G., Gopaluni S., Urban M.L., Gianfreda D., Cassia M.A., Vercelloni P.G., Calatroni M., Bettiol A., Esposito P., Murtas C., Alberici F., Maritati F., Manenti L., Palmisano A., Emmi G., Romagnani P., Moroni G., Gregorini G., Sinico R.A., Jayne D.R.W., Vaglio A. (2020 Sep 6). Slowly progressive anti-neutrophil cytoplasmic antibody-associated renal vasculitis: clinico-pathological characterization and outcome. Clin Kidney J.

[40] Sebastiani M., Luppi F., Sambataro G., Castillo Villegas D., Cerri S., Tomietto P., Cassone G., Bocchino M., Atienza-Mateo B., Cameli P., Moya Alvarado P., Faverio P., Bargagli E., Vancheri C., Gonzalez-Gay M.A., Clini E., Salvarani C., Manfredi A. (2021 Jun 9). Interstitial lung disease and anti-myeloperoxidase antibodies: not a simple association. J. Clin. Med..

[41] Yamaguchi K., Yamaguchi A., Itai M., Onuki Y., Shin Y., Uno S., Hanazato C., Taguchi K., Umetsu K., Aikawa M., Kouno S., Takemura M., Hara K., Motegi S., Tsukida M., Ota F., Tsukada Y., Motegi M., Nakasatomi M., Sakairi T., Ikeuchi H., Kaneko Y., Hiromura K., Maeno T. (2021 Sep). Interstitial lung disease with myeloperoxidase-antineutrophil cytoplasmic antibody-associated vasculitis in elderly patients. Rheumatol. Int..

[42] Fernandez Casares M., Gonzalez A., Fielli M., Caputo F., Bottinelli Y., Zamboni M. (2015 Jul). Microscopic polyangiitis associated with pulmonary fibrosis. Clin. Rheumatol..

[43] Huang H., Wang Y.X., Jiang C.G., Liu J., Li J., Xu K., Xu Z.J. (2014 Jan 28). A retrospective study of microscopic polyangiitis patients presenting with pulmonary fibrosis in China. BMC Pulm. Med..

[44] Arulkumaran N., Periselneris N., Gaskin G., Strickland N., Ind P.W., Pusey C.D., Salama A.D. (2011 Nov). Interstitial lung disease and ANCA-associated vasculitis: a retrospective observational cohort study. Rheumatology.

[45] Hervier B., Pagnoux C., Agard C., Haroche J., Amoura Z., Guillevin L., Hamidou M.A., French Vasculitis Study Group (2009 Mar). Pulmonary fibrosis associated with ANCA-positive vasculitides. Retrospective study of 12 cases and review of the literature. Ann. Rheum. Dis..

[46] Foulon G., Delaval P., Valeyre D., Wallaert B., Debray M.P., Brauner M., Nicaise P., Cadranel J., Cottin V., Tazi A., Aubier M., Crestani B. (2008 Oct). ANCA-associated lung fibrosis: analysis of 17 patients. Respir. Med..

[47] Sun X., Peng M., Zhang T., Li Z., Song L., Li M., Shi J. (2021 Mar 16). Clinical features and long-term outcomes of interstitial lung disease with anti-neutrophil cytoplasmic antibody. BMC Pulm. Med..

[48] Yen C.L., Tian Y.C., Wu H.H., Tu K.H., Liu S.H., Lee C.C., Fang J.T., Yang C.W., Li Y.J. (2019 Oct). High anti-neutrophil cytoplasmic antibody titers are associated with the requirement of permanent dialysis in patients with myeloperoxidase-ANCA-associated vasculitis. J. Formos. Med. Assoc..

[49] Kuligowski M.P., Kwan R.Y., Lo C., Wong C., James W.G., Bourges D., Ooi J.D., Abeynaike L.D., Hall P., Kitching A.R., Hickey M.J. (2009 Jun 18). Antimyeloperoxidase antibodies rapidly induce alpha-4-integrin-dependent glomerular neutrophil adhesion. Blood.

[50] Michailidou D., Mustelin T., Lood C. (2020 Dec 17). Role of neutrophils in systemic vasculitides. Front. Immunol..

[51] Anders H.J., Kitching A.R., Leung N., Romagnani P. (2023 Jul). Glomerulonephritis: immunopathogenesis and immunotherapy. Nat. Rev. Immunol..

[52] Masuda S., Nonokawa M., Futamata E., Nishibata Y., Iwasaki S., Tsuji T., Hatanaka Y., Nakazawa D., Tanaka S., Tomaru U., Kawakami T., Atsumi T., Ishizu A. (2019 Apr). Formation and disordered degradation of neutrophil extracellular traps in Ne-crotizing lesions of anti-neutrophil cytoplasmic antibody-associated vasculitis. Am. J. Pathol..

[53] Yoshida M., Yamada M., Sudo Y., Kojima T., Tomiyasu T., Yoshikawa N., Oda T., Yamada M. (2016 Jul). Myeloperoxidase an-ti-neutrophil cytoplasmic antibody affinity is associated with the formation of neutrophil extracellular traps in the kid-ney and vasculitis activity in myeloperoxidase anti-neutrophil cytoplasmic antibody-associated microscopic polyan-giitis. Nephrology.

[54] Bruner B.F., Vista E.S., Wynn D.M., James J.A. (2011 Jun). Epitope specificity of myeloperoxidase antibodies: identification of candidate human immunodominant epitopes. Clin. Exp. Immunol..

[55] Roth A.J., Ooi J.D., Hess J.J., van Timmeren M.M., Berg E.A., Poulton C.E., McGregor J., Burkart M., Hogan S.L., Hu Y., Winnik W., Nachman P.H., Stegeman C.A., Niles J., Heeringa P., Kitching A.R., Holdsworth S., Jennette J.C., Preston G.A., Falk R.J. (2013 Apr). Epitope specificity determines pathogenicity and detectability in ANCA-associated vasculitis. J. Clin. Invest..

[56] Gou S.J., Xu P.C., Chen M., Zhao M.H. (2013). Epitope analysis of anti-myeloperoxidase antibodies in patients with AN-CA-associated vasculitis. PLoS One.

[57] Free M.E., Stember K.G., Hess J.J., McInnis E.A., Lardinois O., Hogan S.L., Hu Y., Mendoza C., Le A.K., Guseman A.J., Pilkinton M.A., Bortone D.S., Cowens K., Sidney J., Karosiene E., Peters B., James E., Kwok W.W., Vincent B.G., Mallal S.A., Jennette J.C., Ciavatta D.J., Falk R.J. (2020 Jan). Restricted myeloperoxidase epitopes drive the adaptive immune response in MPO-ANCA vascu-litis. J. Autoimmun..

[58] Bossuyt X., Cohen Tervaert J.W., Arimura Y., Blockmans D., Flores-Suárez L.F., Guillevin L., Hellmich B., Jayne D., Jennette J.C., Kallenberg C.G.M., Moiseev S., Novikov P., Radice A., Savige J.A., Sinico R.A., Specks U., van Paassen P., Zhao M.H., Rasmussen N., Damoiseaux J., Csernok E. (2017 Nov). Position paper: revised 2017 international consensus on testing of ANCAs in granulomatosis with polyangiitis and microscopic polyangiitis. Nat. Rev. Rheumatol..

[59] Casal Moura M., Specks U., Tehranian S., Sethi S., Zubidat D., Nardelli L., Dos Santos F.G., Sousa C., León-Róman J., Bobart S.A., Greene E., Zand L., Fervenza F.C. (2023 Jan 1). Maintenance of remission and risk of relapse in myeloperoxidase-positive ANCA-associated vasculitis with kidney involvement. Clin. J. Am. Soc. Nephrol..

[60] Chen M., Xing G.Q., Yu F., Liu G., Zhao M.H. (2009 Apr). Complement deposition in renal histopathology of patients with AN-CA-associated pauci-immune glomerulonephritis. Nephrol. Dial. Transplant..

[61] Villacorta J., Diaz-Crespo F., Acevedo M., Guerrero C., Campos-Martin Y., García-Díaz E., Mollejo M., Fernandez-Juarez G. (2016 Oct). Glomerular C3d as a novel prognostic marker for renal vasculitis. Hum. Pathol..

[62] Oba R., Kanzaki G., Sasaki T., Okabayashi Y., Haruhara K., Okabe M., Yokote S., Koike K., Hirano K., Okonogi H., Tsuboi N., Yokoo T. (2021 Aug 12). Long-term renal survival in antineutrophil cytoplasmic antibody-associated glomerulonephritis with complement C3 deposition. Kidney Int Rep.

[63] Thurman J.M., Frazer-Abel A., Holers V.M. (2017 Nov). The evolving landscape for complement therapeutics in rheumatic and autoimmune diseases. Arthritis Rheumatol..

[64] Xiao H., Schreiber A., Heeringa P., Falk R.J., Jennette J.C. (2007). Alternative complement pathway in the pathogenesis of disease mediated by anti-neutrophil cytoplasmic autoantibodies. Am. J. Pathol..

[65] Lin W., Shen C., Zhong Y., Ooi J.D., Eggenhuizen P., Zhou Y.O., Luo H., Huang J., Chen J.B., Wu T., Meng T., Xiao Z., Ao X., Peng W., Tang R., Yin H., Xiao X., Zhou Q., Xiao P. (2021 Mar 25). Glomerular immune deposition in MPO-ANCA associated glomerulonephritis is associated with poor renal survival. Front. Immunol..

[66] Hilhorst M., van Paassen P., van Rie H., Bijnens N., Heerings-Rewinkel P., van Breda Vriesman P., Cohen Tervaert J.W., Limburg Renal Registry (2017 Aug 1). Complement in ANCA-associated glomerulonephritis. Nephrol. Dial. Transplant..

[67] Sethi S., Zand L., De Vriese A.S., Specks U., Vrana J.A., Kanwar S., Kurtin P., Theis J.D., Angioi A., Cornell L., Fervenza F.C. (2017 Jan 1). Complement activation in pauci-immune necrotizing and crescentic glomerulonephritis: results of a proteomic analysis. Nephrol. Dial. Transplant..

[68] Sethi A., Grande J., Specks U., Fervenza F.C. (2023 Feb 16). Proteomic profile of uninvolved versus crescentic glomeruli in MPO-ANCA-associated vasculitis. Clin Kidney J.

[69] Schreiber A., Xiao H., Jennette J.C., Schneider W., Luft F.C., Kettritz R. (2009 Feb). C5a receptor mediates neutrophil activation and ANCA-induced glomerulonephritis. J. Am. Soc. Nephrol..

[70] Dick J., Gan P.Y., Ford S.L., Odobasic D., Alikhan M.A., Loosen S.H., Hall P., Westhorpe C.L., Li A., Ooi J.D., Woodruff T.M., Mackay C.R., Kitching A.R., Hickey M.J., Holdsworth S.R. (2018 Mar). C5a receptor 1 promotes autoimmunity, neutrophil dysfunction and injury in experimental anti-myeloperoxidase glomerulonephritis. Kidney Int..

[71] Bekker P., Dairaghi D., Seitz L., Leleti M., Wang Y., Ertl L., Baumgart T., Shugarts S., Lohr L., Dang T., Miao S., Zeng Y., Fan P., Zhang P., Johnson D., Powers J., Jaen J., Charo I., Schall T.J. (2016 Oct 21). Characterization of pharmacologic and pharmacokinetic prop-erties of CCX168, a potent and selective orally administered complement 5a receptor inhibitor, based on preclinical evaluation and randomized phase 1 clinical study. PLoS One.

[72] Jayne D.R.W., Bruchfeld A.N., Harper L., Schaier M., Venning M.C., Hamilton P., Burst V., Grundmann F., Jadoul M., Szombati I., Tesař V., Segelmark M., Potarca A., Schall T.J., Bekker P., CLEAR Study Group (2017 Sep). Randomized trial of C5a receptor inhib-itor avacopan in ANCA-associated vasculitis. J. Am. Soc. Nephrol..

[73] Jayne D.R.W., Merkel P.A., Schall T.J., Bekker P., ADVOCATE Study Group (2021 Feb 18). Avacopan for the treatment of AN-CA-associated vasculitis. N. Engl. J. Med..

[74] Heeringa P., Foucher P., Klok P.A., Huitema M.G., Tervaert J.W., Weening J.J., Kallenberg C.G. (1997 Jul). Systemic injection of products of activated neutrophils and H2O2 in myeloperoxidase-immunized rats leads to necrotizing vasculitis in the lungs and gut. Am. J. Pathol..

[75] Foucher P., Heeringa P., Petersen A.H., Huitema M.G., Brouwer E., Tervaert J.W., Prop J., Camus P., Weening J.J., Kallenberg C.G. (1999 Sep). Antimyeloperoxidase-associated lung disease. An experimental model. Am. J. Respir. Crit. Care Med..

[76] Heeringa P., Brouwer E., Klok P.A., Huitema M.G., van den Born J., Weening J.J., Kallenberg C.G. (1996 Nov). Autoantibodies to myelop-eroxidase aggravate mild anti-glomerular-basement-membrane-mediated glomerular injury in the rat. Am. J. Pathol..

[77] Yamakawa H., Toyoda Y., Baba T., Kishaba T., Fukuda T., Takemura T., Kuwano K. (2022 Jul 1). Anti-inflammatory and/or an-ti-fibrotic treatment of MPO-ANCA-positive interstitial lung disease: a short review. J. Clin. Med..

[78] Turgeon D., Balter M.S., Pagnoux C. (2023 Jul 3). Interstitial lung disease in patients with anti-neutrophil cytoplasm anti-body-associated vasculitis: an update on pathogenesis and treatment. Curr. Opin. Pulm. Med..

[79] Yates M., Watts R., Bajema I., Cid M., Crestani B., Hauser T., Hellmich B., Holle J., Laudien M., Little M.A., Luqmani R.A., Mahr A., Merkel P., Mills J., Mooney J., Segelmark M., Tesar V., Westman K.W.A., Vaglio A., Yalçındağ N., Jayne D.R., Mukhtyar C. (2017). Validation of the EULAR/ERA-EDTA recommendations for the management of ANCA-associated vasculitis by disease content experts. RMD Open.

