# Supplementary material for: Presentation and progression of MPO-ANCA interstitial lung disease
Source: J Transl Autoimmun. 2024 Feb 23;8:100235. doi: 10.1016/j.jtauto.2024.100235 (PMC10912625; doi:10.1016/j.jtauto.2024.100235)
Supplement: Multimedia component 1 [file mmc1.docx]

**Appendix A. Supplementary Data**

**Supplement to:** Salvati L, Palterer B, Lazzeri E, Vivarelli E, Amendola M, Allinovi M, Caroti L, Mazzoni A, Lasagni L, Emmi G, Cavigli E, Del Carria M, Di Pietro L, Scavone M, Cammelli D, Lavorini F, Tomassetti S, Rosi E, Parronchi P. Presentation and progression of MPO-ANCA interstitial lung disease. Journal of Translational Autoimmunity, 2024. DOI: 10.1016/j.jtauto.2024.100235

This appendix has been provided by the authors to give readers additional information about the work.

**Supplementary Table 1.** Features of 14 patients with MPO-ANCA ILD.

**Supplementary Table 2.** Results of direct immunofluorescence assay on the kidney biopsies of 6 patients with MPO-ANCA ILD.

**Supplementary Table 1.** Features of 14 patients with MPO-ANCA ILD.

| Case ID | Gender/Age (years) | Ethnicity | Smoking | Pack-years | Stopped smoking (years before first visit) | Silica exposure | Asbestos exposure | Other exposure | Family history of ILD | Anti-MPO abs (UI/ml) at lung disease onset | Age at pulmonary symptoms/signs onset (years) | Symptoms/signs at lung disease onset | ILD pattern on HRCT | Lung biopsy | Age at renal symptoms/signs onset (years) | Renal biopsy | Renal involvement | Time of onset (L=lung; K=kidney) | Time from lung to kidney disease (months) | Treatment | Follow-up from first visit (status) | Follow-up from first visit (years) |
| --- | --- | --- | --- | --- | --- | --- | --- | --- | --- | --- | --- | --- | --- | --- | --- | --- | --- | --- | --- | --- | --- | --- |
| 1 | F/70 | C | No | - | - | No | No | No | No | 17 | 67 | Mild exertional dyspnoea, dry cough | UIP | No | 71 | Yes | Extracapillary proliferative glomerulonephritis | LthenK | 42 | PDN + pirfenidone | On | 5.9 |
| 2 | M/70 | C | Ex | 15 | 42 | No | No | No | No | 14 | 68 | Exertional dyspnoea, dry cough | UIP | No | 68 | Yes | Glomerulonephritis, mixed class (Berden) | LandK | 0 | PDN + RTX + MMF | Deceased | 3.6 |
| 3 | M/73 | C | Ex | 40 | 25 | No | Yes | No | No | 304 | 72 | Worsening dyspnoea, morning dry cough | UIP | No | 71 | No | Proteinuria | KthenL | -13 | PDN + MMF + nintedanib | LTFU | 0.1 |
| 4 | F/74 | C | Ex | 50 | 4 | No | No | No | No | 5,7 | 69 | Worsening dyspnoea, asthenia | NSIP | No | - | No | No | noK | - | TBI | LTFU | 0.1 |
| 5 | F/58 | C | Ex | 11 | 10 | No | No | Parrots | No | 11 | 57 | Exertional dyspnoea | NSIP | Yes | - | No | No | noK | - | MMF | Deceased | 4.7 |
| 6 | M/59 | C | Ex | 40 | 7 | No | No | No | No | 60 | 54 | Worsening dyspnoea | UIP | No | - | No | No | noK | - | LT | Deceased | 4.8 |
| 7 | F/57 | C | Ex | 23 | 2,5 | No | No | No | Yes | 408 | 54 | Worsening dyspnoea, dry cough | Possible UIP | No | 57 | Yes | Necrotizing extracapillary glomerulonephritis, mixed class (Berden) | LthenK | 34 | PDN + MMF + RTX + pirfenidone | On | 3.3 |
| 8 | F/71 | C | No | - | - | No | No | No | No | 32 | 67 | Exertional dyspnoea | UIP | No | 68 | Yes | Extracapillary glomerulonephritis, sclerotic class (Berden) | LthenK | 13 | PDN + MMF + RTX | On | 7 |
| 9 | F/78 | C | No | - | - | No | No | No | No | 8,3 | 76 | Dry cough | Bronchiolitis | No | - | No | No | noK | - | BFI | LTFU | 0.2 |
| 10 | F/72 | C | Ex | 1 | 52 | No | No | No | No | 1,2 | 70 | Exertional dyspnoea | UIP | No | - | No | No | noK | - | MPDN | On | 5.2 |
| 11 | F/64 | C | Ex | 8 | 1 | No | No | No | Yes | 111 | 53 | Incidental, mother with pulmonary fibrosis | UIP | No | 64 | Yes | Glomerulonephritis, focal class (Berden) | LthenK | 125 | PDN + MMF + pirfenidone | On | 3.6 |
| 12 | M/67 | C | Ex | 1 | 50 | No | No | No | No | 53 | 65 | Incidental, abdominal pain (CT of the abdomen performed for a lower back pain) | NSIP | No | 67 | No | Proteinuria | LthenK | 18 | PDN + MMF | On | 6 |
| 13 | M/48 | C | Ex | 7 | 14 | No | No | No | No | 37 | 48 | Dry cough | Bronchiolitis | Yes | - | No | No | noK | - | MPDN + MMF | On | 5.4 |
| 14 | M/60 | C | Ex | 15 | 5 | No | No | No | Yes | 473 | 50 | Dyspnoea, dry cough | UIP | Yes | 55 | Yes | Extracapillary glomerulonephritis | LthenK | 66 | PDN + MPDN + RTX + pirfenidone | Deceased | 4.1 |

BFI = beclometasone/formoterol inhalation; C = Caucasian; ILD= interstitial lung disease; MPDN = methylprednisolone; PDN = prednisone; LT = lung transplant; LTFU = lost to follow-up;

MMF = mycophenolate mofetil; NSIP = nonspecific interstitial pneumonia; RTX = rituximab; TBI = tiotropium bromide inhalation; UIP = usual interstitial pneumonia

**Supplementary Table 2.** Results of direct immunofluorescence assay on the kidney biopsies of 6 patients with MPO-ANCA ILD.

| Case ID | C3  glomerular  parietal desposits | C3  vessel deposits | C4  glomerular  parietal desposits | IgG  glomerular  parietal desposits | IgA  glomerular  parietal desposits | IgM glomerular  parietal desposits | Fibrinogen glomerular  parietal desposits | κ chains glomerular  parietal desposits | λ chains parietal desposits |
| --- | --- | --- | --- | --- | --- | --- | --- | --- | --- |
| 1 | +++ | + | 0 | ++ | ++ | 0 | 0 | ++ | ++ |
| 2 | ++ | + | 0 | 0 | 0 | 0 | 0 | 0 | 0 |
| 7 | + | + | 0 | + | +/- | +/- | 0 | 0 | 0 |
| 8 | + | + | 0 | 0 | 0 | 0 | 0 | 0 | 0 |
| 11 | +/- | + | 0 | 0 | 0 | +/- | 0 | 0 | 0 |
| 14 | ++ | +/- | 0 | + | +/- | ++ | + | ++ | ++ |
